# Experience of Chronic Kidney Disease and Perceptions of Transplantation by Sex

**DOI:** 10.1001/jamanetworkopen.2024.24993

**Published:** 2024-07-31

**Authors:** Latame Komla Adoli, Arnaud Campeon, Valérie Chatelet, Cécile Couchoud, Thierry Lobbedez, Florian Bayer, Elsa Vabret, Eric Daugas, Cécile Vigneau, Jean-Philippe Jais, Sahar Bayat-Makoei

**Affiliations:** 1 Univ Rennes, EHESP, CNRS, INSERM, Arènes – UMR 6051, RSMS – U1309, Rennes, France; 2 Arènes–UMR 6051, ISSAV, EHESP, CNRS, Rennes, France; 3 U1086 INSERM, Anticipe, Centre De Lutte Contre Le Cancer François Baclesse, Centre Universitaire Des Maladies Rénales, Caen, France; 4 Renal Epidemiology and Information Network (REIN) Registry, Biomedecine Agency, Saint-Denis-La-Plaine, France; 5 Inserm U1149 Université Paris Cité Assistance Publique-Hôpitaux De Paris Service De Néphrologie Hôpital Bichat- Paris, Paris, France; 6 Univ Rennes, CHU Rennes, INSERM, EHESP, IRSET (Institut de Recherche en Santé, Environnement et Travail) – UMR_S 1085, Rennes, France; 7 Unité de Biostatistique, Hôpital Necker-Enfants Malades, AP-HP; Institut Imagine; Université Paris-Cité, Paris, France

## Abstract

**Importance:**

The pathway to kidney transplantation (KT) begins with the patient’s acceptance of this surgical procedure after discussion with the nephrologist. The patients’ perceptions of the disease and of KT may influence their willingness to undergo transplantation.

**Objective:**

To describe patients’ experiences of kidney disease and their perceptions of KT and the nephrologists’ perceptions of the patient experience.

**Design, Setting, and Participants:**

This qualitative study collected data through semistructured interviews with patients with chronic kidney disease and nephrologists in the Bretagne, Île-de-France and Normandie regions, France. Researchers involved in the study in each region purposely selected 99 patients with chronic kidney disease who initiated dialysis in 2021, based on their age, sex, dialysis facility ownership, and also 45 nephrologists, based on their sex and years of experience. Data analysis was performed from January to October 2023.

**Main Outcomes and Measures:**

Themes were identified using inductive thematic analysis. Specific characteristics of men and women as well as the nephrologist’s views for each theme were described.

**Results:**

This study included 42 men and 57 women (56 [57%] aged 60 years or older) who started dialysis in 2021 and 45 nephrologists (23 women and 22 men). Six major themes were identified: (1) burden of chronic kidney disease on patients and their families, (2) health care professional–patient relationship and other factors that modulate chronic kidney disease acceptance, (3) dialysis perceived as a restrictive treatment, (4) patients’ representation of the kidney graft, (5) role of past experiences in KT perception, and (6) dualistic perception of KT. In some cases, women and nephrologists indicated that women’s perceptions and experiences were different than men’s; for example, the disease’s psychological impact and the living donor KT refusal were mainly reported by 8 women.

**Conclusions and Relevance:**

Patients’ past experience of chronic kidney disease in general and of KT in particular, as well as their relationship with their family and nephrologist, were substantial determinants of KT perception in this qualitative study. Targeted policies on these different factors might help to improve access to KT, and more research is needed to understand whether there are sex-based disparities.

## Key Points

**Question:**

How do patients receiving dialysis experience chronic kidney disease and perceive kidney transplantation?

**Findings:**

This qualitative study of 99 patients with chronic kidney disease and 45 nephrologists found a dual perception of kidney transplantation: positive because it will put an end to chronic restrictions and negative because it involves uncertainties. Some responses from women and nephrologists indicated that women’s perceptions and experiences were different than men’s.

**Meaning:**

These results suggest that actions on patients’ dialysis experience should be made to improve access to kidney transplantation.

## Teaser

This qualitative study characterizes patient experiences of chronic kidney disease and perceptions of kidney transplantation as well as nephrologists’ perceptions of the patient experience.

## Introduction

For eligible patients, kidney transplantation (KT) is the best kidney replacement therapy to improve symptoms and quality of life.^1,2,3^ However, in many countries, access to KT is a serious issue due to the limited availability of grafts and the high demand.^4^

Previous studies in France and abroad reported the existence of disparities in the access to KT.^5,6,7^ These disparities may be associated with socioeconomic level, ethnicity, the practice of nephrologists, or with sex and/or gender.^8,9,10,11,12,13,14^ Some authors concluded that women refuse transplantation much more than men, whereas they are more likely to be living donors.^15,16^ Patients’ preferences could play an important role in the explanation of these disparities.^15,17^ Hence, it is valuable to understand how these disparities arise and to identify factors on which action could be focused to improve access to KT. In France, according to the Haute Autorité de la Santé (HAS) recommendations, entry into the KT process starts with the proposal by the nephrologist and a discussion with the patient.^18^ After accepting to start the process, the patient will undergo medical tests and see different health care professionals who will confirm the absence of contraindications.

Understanding the patients’ experiences and perceptions of KT will help to address disparities and to adapt policies on KT. Therefore, this study was designed to address the following question: how do patients on dialysis experience chronic kidney disease (CKD) and perceive KT?

## Methods

This qualitative study was part of a larger project which aimed to understand sex-based and/or gender-based disparities of access to KT in France.^19^ This study and the data collection were authorized by the French National School of Public Health data protection officer. All patients and nephrologists read an information letter and gave their informed oral consent prior to the interviews.

### Study Design

A descriptive qualitative study was performed based on interviews with patients and nephrologists.^20,21^ This study was guided by a phenomenological approach and focused on the experience of CKD as perceived by patients and nephrologists.^22,23^ The terms “man/men” and “woman/women” used in this work refer to the biological sex.^24^

### Participants’ Recruitment

Participants were recruited from 3 French regions: Bretagne, Normandie, and Île-de-France. Without seeking exhaustiveness, these regions have different profiles concerning KT access and were chosen to ensure heterogeneity.^25,26,27^

L.K.A. (MD, MSc, man, with experience in quantitative and qualitative studies), S.B. (MD, PhD, woman, with experience in quantitative and qualitative studies) and the researchers involved in the study in each region purposely selected 99 patients with CKD who initiated dialysis in 2021, based on their age, sex, dialysis facility ownership, and also 45 nephrologists, based on their sex and years of experience. Patients with absolute contraindication to KT according to the HAS recommendations (age >85 years, active malignant neoplasm, body mass index [calculated as weight in kilograms divided by height in meters squared] >50, oxygen-dependent) were not included.^18^

### Data Collection

Data were collected using semistructured interviews. F.K. and S.D. (MPH, woman, experts in qualitative and quantitative studies), A.A. (MPH, man, expert in quantitative and qualitative studies) and L.K.A. performed the interviews. They were not involved in the patients’ care and underwent training to homogenize practices before starting the interviews. In collaboration with A.C. (PhD, man, senior researcher in qualitative studies), L.K.A. elaborated a patient and nephrologist interview guide that was pretested with patients and nephrologists. This guide listed the themes covered during the interviews using open-ended questions. These themes were identified from the literature^28,29,30,31,32^ and based on the experience of the researchers involved in this study (eAppendix 1 and eAppendix 2 in Supplement 1). The interviews were carried out face-to-face, in French, at the hospital during a dialysis session (with preservation of confidentiality) or at the patient’s home in a quiet place. All interviews were recorded on a dictaphone. Field notes were made during interviews.

### Data Analysis

The interviewers or a transcription agency transcribed the interviews. The Nvivo software (version 1.7.1, QSR International)^33^ was used to code the data. L.K.A. and S.B. independently coded the data using an inductive broad-based approach. Then, a thematic analysis was used to group codes into common themes. The interviews of patients and nephrologists were analyzed together. First, each theme was identified and then the specific characteristics of men and women as well as the nephrologist’s views for each theme were pointed out. A.C. reviewed independently the data and the codes to contribute to the analysis. This study followed the Consolidated Criteria for Reporting Qualitative Research (COREQ) framework to ensure that the important aspects of this study were reported.^34^ Saturation was reached when the analysis did not bring any new additional theme.^35^ However, interviews were continued and all interviews were analyzed to ensure that no new element would emerge. Five patients received their interview transcript to confirm it. The results of the analysis were presented to 2 patients and 5 nephrologists to obtain their feedback. They did not suggest any change in the quotes. Ten patients refused to take part in this study for various reasons.

## Results

### Patients’ Characteristics

For this study, 99 patients (57 women, 42 men, and 56 [57%] aged 60 years or older) and 45 nephrologists (23 women and 22 men) were interviewed (Table 1). Interviews lasted approximately 45 minutes.

**Table 1.** Characteristics of Study Participants

Characteristics	Patients, No. (%)			Nephrologists, No. (%)		
	Men (n = 42)	Women (n = 57)	Total (N = 99)	Men (n = 22)	Women (n = 23)	Total (N = 45)
Region of residence						
Bretagne	4 (9)	11 (19)	15 (15)	7 (32)	8 (35)	15 (33)
Île-de-France	18 (43)	26 (46)	44 (45)	8 (36)	7 (30)	15 (33)
Normandie	20 (48)	20 (35)	40 (40)	7 (32)	8 (35)	15 (33)
Age group, y						
<60	16 (38)	27 (47)	43 (43)	NA	NA	NA
≥60	26 (62)	30 (53)	56 (57)	NA	NA	NA
Time working as nephrologist, y						
<10	NA	NA	NA	9 (41)	15 (65)	24
≥10	NA	NA	NA	13 (59)	8 (35)	21

Abbreviation: NA, not applicable.

### Interview Themes and Subthemes

The thematic analysis of the interviews identified 6 main themes: (1) burden of chronic kidney disease on patients and their families, (2) health care professional-patient relationship and other factors that modulate CKD acceptance, (3) dialysis perceived as a restrictive treatment, (4) patients’ representation of the kidney graft, (5) role of past experiences in KT perception, and (6) dualistic perception of KT. Table 2 lists selected illustrative quotations to support each theme and the Figure presents a graphical synthesis of the analysis results.

**Table 2.** Selected Illustrative Quotations

Themes	Examples of quotes
**Theme 1: Burden of chronic kidney disease on patients and their families**	
Physical and psychological impact of CKD on patients’ daily life	“We don’t have the energy; we don’t have the... we’re tired all the time. We feel diminished, we realize, yeah.” (patient 1, more than 60 years of age, woman)
	“... it’s as if we’re going to give up something. And the hyperactivity in our head is ‘I can’t be tired, it’s not possible. It’s not possible.’ And yet our body tells us ‘Hey, you’re going to rest a little here because this is not going to work.” (patient 2, less than 60 years of age, woman)
	“So, your self-image is... it’s true that it’s very damaged in fact, because on top of that you’ve got a foreign body, an ugly thing.” (patient 3, more than 60 years of age, woman)
	“Men find it harder to express their fears than women.” (nephrologist 4, man)
	“I could have the impression that men are more stressed than women, but I’m not sure.” (nephrologist 5, man)
Impact of CKD on patients’ relationship with their family	“And now I think... I don’t know how to explain it, well I think we still love each other, but we don’t have any... it’s been two and a half years since we’ve had a sexual relationship, we’re no longer a couple in that sense.” (patient 3, more than 60 years of age, woman)
	“So, spontaneously, I’d tend to think that women are a little more preoccupied than men. But it’s not very significant.” (nephrologist 1, man)
	“… in fact, because of this, my husband left me [laughs]. When the dialysis was approaching, he told me he’d had enough of living with a patient and that was that.” (patient 40, less than 60 years of age, woman)
**Theme 2: Healthcare professional-patient relationship and other factors that modulate CKD acceptance**	
Good relationship with health care professionals	“[Laughs]. It’s really night and day. No, I’ve been lucky, I’m happy here, we’re well looked after, we’re well looked after. If you have the slightest health problem, you talk to... you need to talk to the nephrologists, they take the initiative right away, they make appointments for you left and right. They put me at ease, frankly.” (patient 4, more than 60 years of age, man)
	“But that’s good because it’s in addition, because it allows us to learn much about dialysis, how to make the connections for the prep (preparation) and everything, eh? I already know a bit about it. But it’s important anyway, and it allows us to talk to the doctors and all that.” (patient 5, more than 60 years of age, woman)
Difficult relationship with health care professionals	“The nephrologist, I thought, wasn’t... he wasn’t expressing himself, because he said I was a patient who asked too many questions. Not so long ago, when he was treating people, people didn’t ask so many questions.... But we didn’t agree, so there you go.” (patient 6, less than 60 years of age, woman)
	“Men are more likely to disagree and to want to force... women, I have the impression, are more trusting and better accept my explanations and advice.” (nephrologist 1, man)
	“And I noticed that. So, we’re numbers, we’re customers.” (patient 7, more than 60 years of age, woman)
	“And they’re... we go to hospital, they’re not friendly, they’re unpleasant.” (patient 32, more than 60 years of age, woman)
Role of family and friends in coping with the disease	“They supported me. From beginning to end, my husband is very, very present. He’s the one who prepares my dialysis every day.” (patient 8, less than 60 years of age, woman)
	“As for patients, yes, I find that women take things more seriously. They’re less likely to be accompanied by their husbands (nephrologist 11, woman).”
Role of religious beliefs in CKD acceptance	“Yes. But... me, you know, I’m a Christian. I’m a believer and... I’m not afraid of that, I’m not afraid of death. I’m not afraid of it, because I have faith.... Yes, that’s it. And it’s all thanks to God, I say ‘That’s the way it is.’ Even if I’m on dialysis, we can still live, like everyone else [laughs].” (patient 9, less than 60 years of age, woman)
	“It’s no big deal. And uh… no, no, I took it upon myself and that’s it, I trust in God because I’m a religious person. And that helped me a lot.” (patient 15, less than 60 years of age, woman)
**Theme 3: Dialysis perceived as a restrictive treatment**	
Different perception of dialysis in function of the initiation mode	“Everyone explained it to me, but it was all in my head. It was... I couldn’t... I couldn’t get used to the idea that I was going to start dialysis soon....” (patient 5, greater than 60 years of age, woman)
	“In general, patients we’ve been following for a long time, what we call CKD (chronic kidney disease), are already aware of what’s going on. So psychologically, they take it fairly well, because they’re prepared for consultations with the doctor, dietician and nurses. After that, the others are very heterogeneous. Some patients take it very well, others very badly. It’s very heterogeneous.” (nephrologist 2, man)
	“We knew about the disease in the family. So, I knew that sooner or later, I was going to start dialysis.” (patient 10, less than 60 years of age, man)
Adaptation to dialysis and its impact on patient health	“With dialysis and all that, my health is improving. Even my legs are less swollen, so that’s good. Well, it means a lot to me. Because now I can do little things that before I couldn’t, but now I can.” (patient 11, greater than 60 years of age, woman)
	“Before dialysis, I had trouble breathing. But after dialysis, frankly, there’s a lot of improvement. I feel like I’m 20 again, as they say. That’s how it is. It’s a bit tiring. Sometimes it’s a bit tiring. But it’s all right. The only thing that bothers me about dialysis is the fistula.” (patient 33, less than 60 years of age, man)
**Theme 4: Patients’ representation of the kidney graft**	
A gift or a bequest for a normal life	“It’s still [gesture for “more”], yes that’s it. He’s giving me something that’s more expensive than life, and it’s going to be even more expensive for me, and I have to take care of it even more.” (patient 12, greater than 60 years of age, woman)
	“I am telling you yes, it is a bump in the road of my life that it is in the process to be repaired and that will be completely repaired once I’ll have my graft. An it will be my job to look after this graft because it is a life gift.” (patient 2, less than 60 years of age, woman)
A foreign object	“But a transplant, for me no matter what, is a foreign element.” (patient 13, greater than 60 years of age, man)
	“Are we going to... a foreign body, it might feel weird. That’s the kind of apprehension we have, but then, fear, no.” (patient 14, less than 60 years of age, man)
	“There have been very few patients who have in fact... to have an organ from someone else, who have told me that it couldn’t be possible. There were perhaps two who told me that they couldn’t have an organ that wasn’t theirs.” (nephrologist 13, man)
The graft origin (living or deceased donor) as a determinant of its representation	“For me, it’s very selfish.... And if I take the kidney of a living person and that person has a problem tomorrow and needs both kidneys, what shall I do? What shall I do?” (patient 15, less than 60 years of age, woman)
	“Not at my age. And then with everything you have to do... No, no. My daughter wanted to give me her kidney and I said, ‘No, I don’t want it.’ I said, ‘You never know, you’ve got 3 kids, you never know what might happen.’ And that doesn’t mean it [the graft] is going to work.” (patient 16, greater than 60 years of age, woman)
	“Maybe the only sex difference I see in my experience, but I haven’t either.... Well, thousands of cases like that. Maybe it’s with regard to donations from a child, that is to say, maybe [stresses the “maybe”] dads tend to accept kidney donations from a child more than moms.” (nephrologist 3, woman)
	“He talked to me, yes, but I don’t have anyone for that, me [laughter]. It’s not easy, no one accepts, when you talk to them about it, they’ll say ahh, you know, not now. They don’t know you can live with only one kidney.” (patient 17, less than 60 years of age, woman)
	“No, because I don’t intend to ask my family, I don’t think it’s up to us to ask for a donation, it has to come from the person, brothers and sisters what, I haven’t asked for anything.” (patient 6, less than 60 years of age, woman)
**Theme 5: Role of past experiences in KT perception**	
Dialysis experience in the construction of KT perception	“Dialysis is very heavy, it takes up your life. But I’m lucky because dialysis has always gone well. I don’t have any drawbacks and the results are just about right. So, I’ve always been afraid that the transplant wouldn’t go well. I’ve always been afraid that the transplant would fail or that I’d die.” (patient 18, greater than 60 years of age, woman)
	“You have a transplant and then you stop dialysis to begin with, automatically, because dialysis is a handicap. It’s three times per week and blocked for four hours, so it’s a handicap for life all the same.” (patient 36, greater than 60 years of age, man)
Personal and family experience of KT	“I know someone who’s had 3 transplants and they didn’t work. So, given what I have, no.” (patient 25, greater than 60 years of age, man)
	“Well, after the transplant, they say it takes a long time to get through it, but it’s worth it anyway. That’s why I want to go all the way.” (patient 34, less than 60 years of age, woman)
	“There’s my mother who has an uncle who had a transplant, but we don’t have a direct link. As a result, he already had a transplant and it was... he had the same graft for 20 years, so it went well for him, and that’s that.” (patient 35, less than 60 years of age, man)
**Theme 6: Dualistic perception of KT**	
Breaking free from chronic suffering and resuming normal life	“After the transplant... once you’ve been transplanted, hop! You go back to your normal life.” (patient 19, greater than 60 years of age, woman)
	“In any case, they see a transplant as a solution when you talk to them about it.” (nephrologist 14, man)
	“For me, a transplant is a wonderful thing [laughs]. It’s something that allows you to live again. So, anyway, it’s all positive for me.” (patient 29, less than 60 years of age, woman)
From mistrust to refusal	“... I’m not interested in it, I’m not interested in the transplant. You know, I’m almost 70, I’ve got a lot of problems, I’ve got a lot of things happening to me. So, I don’t know, I could die in two or 3 years, or even sooner, so I’m not interested in a transplant. I’m not going to have fun having a transplant when I’m not sure it’s going to succeed, and there are big problems afterwards too.” (patient 20, greater than 60 years of age, man)
	“And today, in fact, it’s too weird, but I don’t want to have a transplant because I’m scared. For me, it seems to be a process full of dangers.” (patient 27, less than 60 years of age, woman)
	“Yes, I think they do. I think that women are saying to themselves... yes, I think they’re delaying the check-up…. And I think that we women pay more attention to our bodies and perhaps accept less interventions on our bodies than men do.” (nephrologist 10, woman)
	“I’ve had more men who were reluctant to have a transplant than women.” (nephrologist 11, woman)

Abbreviations: CKD, chronic kidney disease; KT, kidney transplantation.

**Figure.**
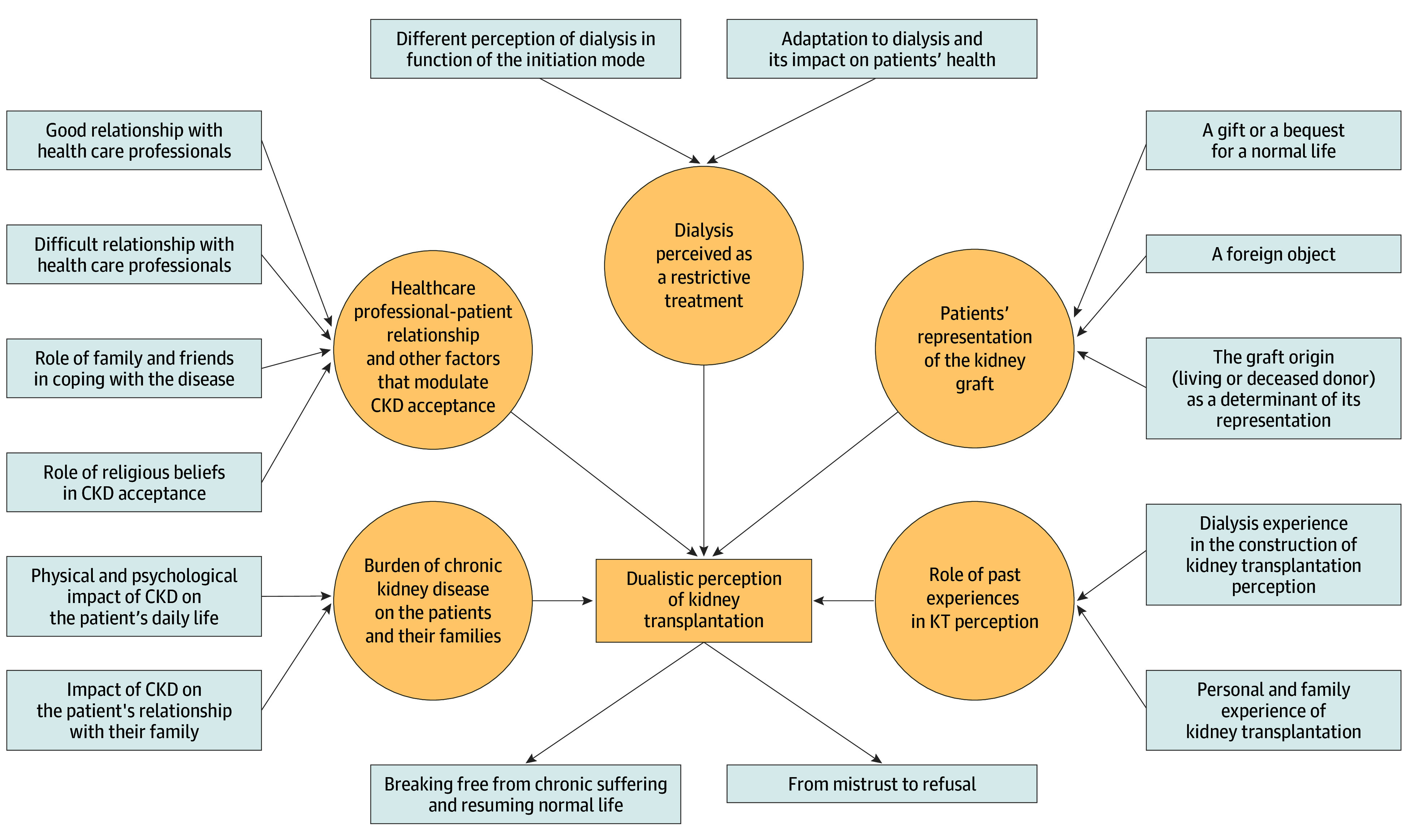
Graphical Synthesis of the Themes and Subthemes Themes are shown in tan and subthemes in light gray. The arrows show how the subthemes relate to the themes and how certain themes relate to each other. CKD indicates chronic kidney disease.

### Theme 1: Burden of Chronic Kidney Disease on Patients and Their Families

#### Physical and Psychological Impact of CKD on Patient’s Daily Life

Patients mentioned the difficulty of living with CKD. CKD was seen as an all-encompassing disease that affected all aspects of the patient’s life (professional, social, personal). Patients stressed the daily fatigue that prevented them from feeling fulfilled. A patient elaborated, “we’re tired all the time” (patient 1, more than 60 years of age, woman). Whatever the patient’s age, the fatigue onset marked the first turning point in the normal course of their life. However, despite all efforts and determination to maintain a life as normal as possible, at some point, the feeling of exhaustion disrupted the patient’s daily routine, from going to work and carrying out professional activities, to more ordinary activities, such as going for a walk, taking part in leisure activities, or even feeling autonomous in the most basic activities of daily living. These limitations and the impossibility of overcoming this fatigue could affect the patients’ self-esteem and mental health. Patients felt that they were not functioning as well as they used to, that their body mechanics had broken down. This feeling was reported frequently by women. A nephrologist elaborated “men find it harder to express their fears than women” (nephrologist 4, man) and “I have the impression that men are more stressed than women, but I’m not sure” (nephrologist 5, man).

#### Impact of CKD on Patient’s Relationship With Family

CKD also affected the patient’s family and close circle of friends. The emotional bond between parent and child, patient and employer, patient and friend, and the couple relationship were especially affected. The impact on the family was positive for several patients, in terms of strengthening the existing relationships or creating new ones. However, some patients experienced difficulties in their relationships with others. A woman younger than 60 years elaborated, “… in fact, because of this, my husband left me [laughs]. When the dialysis was approaching, he told me he’d had enough of living with a patient and that was that” (patient 30). Two women reported the impact on their partner and on their married life. CKD did not allow them to play their full role in the couple, particularly in caring for their partner or contributing to the couple’s sexual fulfilment.

### Theme 2: Health Care Professional–Patient Relationship and Other Factors That Modulate CKD Acceptance

#### Good Relationship With Health Care Professionals

CKD progression and the care pathway of the patients with CKD involve several health care professionals who enter and leave the care pathway at different times. Not surprisingly, several patients said that the quality of the health care professional–patient relationship was very good and was important for their disease management. A patient shared, “… Yes, and the staff members are really nice” (patient 31, more than 60 years of age, man). A good relationship with the staff provided comfort, reassurance and even greater acceptance of the disease and dialysis. A good relationship included a warm welcome, a good explanation of the disease and treatment, a willingness to answer questions, moral and emotional support, and greater patient involvement in decision-making.

Similarly, the dialysis center gradually became more familiar, and was seen less as a place of care and more as a place where they spent a lot of time and where they developed new points of reference. Therapeutic education sessions were also mentioned and helped to establish this close relationship.

#### Difficult Relationship With Health Care Professionals

The health care professional-patient relationship is very important for CKD management. However, this relationship was not always easy for some patients who reported difficulties in communicating with their health care professionals. Some women, reported difficult relationships with their doctors/nephrologists who had a very hierarchical and overbearing behavior toward them. A patient elaborated: “And they’re... we go to hospital, they’re not friendly, they’re unpleasant” (patient 32, more than 60 years of age, woman). This situation, far from being trivial, often led to a deterioration of self-perception in patients who would have needed support. This aspect was not raised by the nephrologists who thought that “men are more likely to disagree and to want to force...” (nephrologist 1, man).

#### Role of Family and Friends in Coping With CKD

Close friends and family played an important role in coping with CKD. Indeed, being surrounded by people who understood the disease and were willing to help improved the patients’ comfort and psychological state, and also enabled them to draw on their resources to remain independent. A nephrologist thought that women were much braver and were “less likely to be accompanied by their husbands” (nephrologist 11, woman).

#### Role of Religious Beliefs in CKD Acceptance

Belonging to a religious faith was an important factor cited by patients as making it easier for them to accept their disease. Illness was seen by them as a divine decision, and faith allowed them to free themselves from the fear of the disease and its consequences. This point was raised by 2 women. Faith gave them a safe space to retreat to and facilitated the acceptance of difficulties and suffering.

### Theme 3: Dialysis Perceived as a Restrictive Treatment

#### Different Perception of Dialysis in Function of the Initiation Mode

Starting dialysis is an important stage in the patient’s life. Initially, it was perceived as a restrictive factor, making a break in the patients’ normal life because their survival now depended on a machine, unlike the previously enjoyed freedom and autonomy. However, this perception was not shared by all patients, especially when transition to dialysis had been planned for a long time. For these patients, there had been a gradual learning process: they had time to prepare themselves mentally for this treatment modality and to accept it as a necessary constraint to improve their health.

#### Adaptation to Dialysis and Its Impact on Patient Health

Once treatment had begun, dialysis benefits were clearly seen, and patients testified that it improved their health and more generally, their quality of life. Accepting dialysis helped to give a new meaning to life, allowing patients to regain a sense of self-confidence in their body, and to resume activities they could no longer do. This positive view of dialysis was more pronounced in patients who experienced very disabling symptoms before dialysis initiation.

### Theme 4: Patients’ Representation of the Kidney Graft

#### A Gift or a Bequest for a Normal Life

The patients’ perceptions of the graft varied. For some, the kidney graft was seen as a gift, a very expensive present that would allow them to have a normal life again. At times, the precious nature of the graft gave rise to a feeling of indebtedness and a promise to take care of it.

#### A Foreign Object

However, the kidney graft was not always well accepted. For some patients, it was a foreign object that alters their body equilibrium and that they were not ready to accept. A man patient shared “But a transplant, for me no matter what, is a foreign element” (patient 13, more than 60 years of age, man).

#### The Graft Origin (Living or Deceased Donor) as a Determinant of Its Representation

Overall, the interviewed patients were in favor of receiving a kidney from a deceased donor; conversely, receiving a kidney from a living donor posed an ethical dilemma that led some patients to refuse it. It seemed difficult not to feel guilty about a possible future deterioration in the donor’s health. This feeling was reported by 8 women.

For patients who were prepared to accept a kidney from a living person, the relationship with the donor was another issue that needed to be tackled. For example, receiving a kidney from one’s child was completely impossible. Indeed, a nephrologist explained that it was more complicated for a mother to receive a kidney from her child, whereas this was not always the case for a father.

A living-donor transplant remained an option to be pursued for some patients. However, this brought new challenges. The first was to find a relative who was willing to donate a kidney, and the second was to ensure that the donor was compatible. Some patients reported difficulties in finding a donor. Poor relationships with family and friends, or lack of understanding by family and friends were all barriers to living-donor transplant. Moreover, some patients said that it was not up to them to ask for a kidney, but that those around them should spontaneously suggest this donation.

### Theme 5: Role of Past Experiences in KT Perception

#### Dialysis Experience in the Construction of KT Perception

When dialysis was going well, and the patient’s health improved, interest in KT was not high. Patients found a certain balance on dialysis and were more concerned about the KT risks and its impact on their health. A patient elaborated: “... I’m stabilizing my life like this. And then, maybe, I’ll want something else. But today, my life like this suits me just fine” (patient 8, less than 60 years of age, woman). This is a patient with a good experience about dialysis and who has the support of her partner and family. Conversely, patients who experienced difficulties with dialysis saw KT as a goal, a redeeming feature, something to help return to normal life.

#### Personal and Family Experience of KT

It was common to ask people who underwent KT what to expect, and whether they felt reassured by their decision. This feedback could be positive, which reassured the patient, or negative, which raised new questions, without dissuading them.

### Theme 6: Dualistic Perception of KT

The patients’ perceptions of KT were a key factor in determining its acceptance. On the one hand, patients viewed KT as an opportunity to resume a normal life; on the other, fear or mistrust were given as reasons to refuse surgery.

#### Breaking Free From Chronic Restrictions and Resuming Normal Life

KT was seen as a break from the difficult, restricted life of dialysis. Patients thought that KT would allow resuming activities, would reduce the dialysis burden. They also saw it as a source of hope for resuming “normal” life. KT was considered an important opportunity.

This vision of KT allowed patients to maintain a positive view of the future. This feeling was reinforced by the positive feedback from close friends and family and by their trust in medicine and health care professionals. However, this positive perception of KT was not shared by all interviewed patients, due to their specific journey.

#### From Mistrust to Refusal

Fear of surgery, uncertainty about the surgery side effects, and fear of the unknown when undergoing transplantation were factors likely to hinder KT acceptance. Some patients felt quite comfortable on dialysis and did not see the need to start a complex process with an uncertain outcome. For these patients, the sacrifice was not worth it, and they preferred to maintain their life balance without undergoing KT. Age could partly explain this lack of commitment to new treatments. Older patients were skeptical about KT. Moreover, some nephrologists thought that women deliberately postponed the start of their pre-KT workup due to fear of what may be found. However, not all nephrologists shared this view “I’ve had more men who were reluctant to have a transplant than women” (woman nephrologist). Women seemed to be as hesitant as men about KT.

## Discussion

The present study investigated what patients with CKD on dialysis thought about KT and how this perception was constructed. This is the first time, to our knowledge, that the question of KT perception has been studied using a qualitative approach and including a large number of patients and nephrologists. Themes included the burden of CKD on patients and their families, health care professional–patient relationship and other factors that modulate CKD acceptance, dialysis perceived as a restrictive treatment, patients’ representation of the kidney graft, role of past experiences in KT perception, and dualistic perception of KT. Our findings suggest there are sex-specific differences in how men and women experience CKD and KT. The feeling of being stripped of their self, the impact of the disease on marital relationships, the difficult relationship with health care professionals, the role of religion in managing the disease, and the refusal of living-donor transplant were reported by a few women patients. Men patients perceived the kidney graft as a foreign object. Some nephrologists thought that women talked frequently about the impact of the disease, whereas men appeared to be much more stressed.

Almost all patients reported the burden of kidney failure. Patients mentioned fatigue as the first symptom, in line with previous studies.^36,37^ Several patients, particularly women, also mentioned the psychological burden of the disease. Past studies reported that women tend to have much more severe symptoms than men and also experience a higher psychological burden.^38,39^ Dialysis onset is the other key stage in CKD management. It is a turning point in the patients’ lives. This study highlighted that patients who have spent a long time monitoring their disease before starting dialysis cope better with it than those who started it as an emergency. This underlines the need for early management before the start of dialysis, with good coordination between the general practitioner and the nephrologist, but also with the involvement of other specialists such as psychologists.^40^

The nature of the health care professional-patient relationship also influenced the disease management. The quality of this relationship is very important, as stated by several patients. It involves listening to the patients, answering to their questions and providing the necessary information. In a previous study in a dialysis center, most patients said that the therapeutic nurse–patient relationship was important in kidney replacement therapy because it gave them the sense to be understood and the strength to fight the disease.^41^ This also implies the patients’ involvement in the decision-making process.^42,43^

Concerning the patients’ perception of KT, it emerged that not all patients perceived KT in the same way. Previous experiences with KT, personal or by a close relative, as well as the risks associated with surgery were some obstacles to KT acceptance identified in our study. The same reasons, including dying after general anesthesia or needing additional surgical interventions, were identified by a narrative review of qualitative studies on access to KT.^44^

For living-donor transplants, the relation with the donor and the graft representation were the main barriers reported. Unlike the present work, a study carried out in the USA on patients’ perceptions of KT showed that living-donor KT was preferred and that the financial burden was an obstacle to KT acceptance.^45^ One explanation for these differences might be that CKD treatment is fully reimbursed by the social security in France.

Most of the identified themes were shared by men and women; however, we have identified a number of points that could suggest that there are differences between men and women. CKD impact on the psychological well-being and on marital relationships was mentioned mainly by women, possibly because women tend to have a worse estimated psychosocial health than men.^46^ We also showed that few women did not want a graft from a living donor, especially when the donor was their child. This refusal was due to different reasons, including not wanting to be the cause of any future deterioration in the donor’s health, and finding this act selfish, especially given the KT uncertain outcome. This is in line with a previous finding that women are less willing to undergo KT compared with men.^15,47,48^ However, some men also refused KT, particularly due to advanced age and the perception of the graft as a foreign element. More studies are needed to confirm these disparities.

### Limitations

This study has limitations. Interviews were carried out by different interviewers. Although they were trained and used the same interview guide, some residual subjectivity inherent to each interviewer remained. Moreover, the effect of the nephrologists’ sex on their reported perceptions was not investigated although it may have influenced their answers. Additionally, this study was carried out in French and then, results and quotes were translated into English. Although the translation was carried out by a specialized translation agency, the meaning of some words and phrases may differ slightly from the French version.

## Conclusions

This qualitative study described the patients’ perceptions and experiences of KT and identified a number of points that could suggest that there are differences between men and women, which could help explain women’s lower access to KT in France. The sex-based differences observed need to be further investigated using a systematic gender analysis.

## Notes

Supplement 1. **eAppendix 1.** Patients’ Interview Guide **eAppendix 2.** Nephrologists’ Interview Guide

Supplement 2.
Data Sharing Statement
