# Supplementary material for: Experience of Chronic Kidney Disease and Perceptions of Transplantation by Sex
Source: JAMA Netw Open. 2024 Jul 31;7(7):e2424993. doi: 10.1001/jamanetworkopen.2024.24993 (PMC11292447; doi:10.1001/jamanetworkopen.2024.24993)
Supplement: Supplement 1. — eAppendix 1. Patients’ Interview Guide eAppendix 2. Nephrologists’ Interview Guide [file jamanetwopen-e2424993-s001.pdf]

Supplemental Online Content

Adoli LK, Campeon A, Chatelet V, et al. Experience of chronic kidney disease and perceptions of transplantation by sex. *JAMA Netw Open.* 2024;7(7):e2424993. doi:10.1001/jamanetworkopen.2024.24993

eAppendix 1. Patients' Interview Guide

eAppendix 2. Nephrologists' Interview Guide

This supplemental material has been provided by the authors to give readers additional information about their work.

© 2024 Adoli LK et al. *JAMA Network Open.*

eAppendix 1. Patients' Interview Guide

- Can you provide me information about yourself (age, education, income, living place, marital status)
- The start of the disease and it course
  - ✓ The first signs
  - ✓ The diagnosis
  - ✓ treatment
  - ✓ Impact
- Perception on the disease
- Information the disease treatment
- Perception on kidney transplantation?
    - Deceased donor
    - Living donor
    - pathway
  - Have you ever attended a group education session with other patients?
  - How do you feel about kidney transplantation?
    - How do you consider transplantation?
    - Did you receive feedback on the advantages/disadvantages of transplantation?
    - How do think a kidney transplant may change your health?
  - Kidney transplantation proposal?
  - Are you in the process of having a pre-transplant work-up?
  - How do you see the future?

© 2024 Adoli LK et al. *JAMA Network Open.*

eAppendix 2. Nephrologist' Interview Guide

- Could you tell me a little bit about your work as a nephrologist?
- Could you describe the first consultation with a patient with CKD (stage 4 and 5) whom you never saw before?
  - ✓ In which circumstances do they come to you?
  - ✓ How are their feelings?
  - ✓ What kind of general information do you give to patients at the first consultation?
- In which context do you discuss the possibility of transplantation with your patients?
- Can you tell me about the information you give to patients about kidney transplantation and how they consider this information?
  - ✓ Do you tailor the information in function of the patient profile, sex?
  - ✓ Do you give information on living donor transplantation?
- Do you suggest other information sources to your patients?
- Do you talk about transplantation with non-transplantable patients?
- Do patients ever refuse to undergo kidney transplantation?
  - ✓ What are the reasons for refusal?
  - ✓ How do you manage patients refusing kidney transplantation?
  - ✓ Is there any particularity according to patient' sex?
- How do you see the collaboration between you and the transplant team?
- We will now talk about the patient pre-transplant work-up. How do the different stages of this work-up take place?
- How do patients (men and women) experience this process?
- What is your opinion about access to kidney transplantation for women in France?
- In your opinion, what should be done to improve the overall access to kidney transplantation in France? And particularly for women?

© 2024 Adoli LK et al. *JAMA Network Open.*
