# Supplementary material for: Experience of Chronic Kidney Disease and Perceptions of Transplantation by Sex
Source: JAMA Netw Open. 2024 Jul 31;7(7):e2424993. doi: 10.1001/jamanetworkopen.2024.24993 (PMC11292447; doi:10.1001/jamanetworkopen.2024.24993)
Supplement: Supplement 2. — Data Sharing Statement [file jamanetwopen-e2424993-s002.pdf]

Data Sharing Statement

Adoli. Experience of Chronic Kidney Disease and Perceptions of Transplantation by Sex. *JAMA Netw Open*. Published July 31, 2024. doi:10.1001/jamanetworkopen.2024.24993

Data
Data available: No

Additional Information
Explanation for why data not available: The data underlying this article cannot be shared publicly for the privacy of individuals that participated in the study.
